# 4-(4-Methylbenzenesulfonamido)benzoic acid *N*,*N*-dimethyl­formamide mono­solvate

**DOI:** 10.1107/S1600536811011949

**Published:** 2011-04-07

**Authors:** Miao-Ling Huang, Zhi-Yang Lin

**Affiliations:** a College of Chemistry and Science of Life, Quanzhou Normal University, Fujian 362000, People’s Republic of China

## Abstract

In the title compound, C_14_H_13_NO_4_S·C_3_H_7_NO, the C—S—N—C torsion angle of −64.55 (17)° defines the folded conformation of the mol­ecule. The dihedral angle between the benzene rings is 83.367 (6)°. In a crystal, mol­ecules are linked into a chain along *a* axis through inter­molecular N—H⋯O and O—H⋯O hydrogen bonds. There is also an intra­molecular C—H⋯π inter­action.

## Related literature

*N*-protected amino acids possess an *R*-CONH-*R*′ group analogous to the structure of *O*-terminal peptides and proteins, see: Antolini *et al.* (1984 ▶); Menabue & Saladini (1988 ▶).
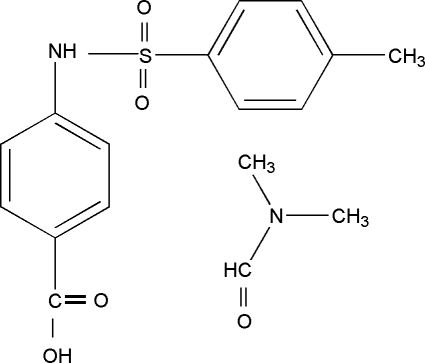


## Experimental

### 

#### Crystal data

C_14_H_13_NO_4_S·C_3_H_7_NO
*M* *_r_* = 364.41
Monoclinic, 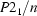

*a* = 8.0953 (10) Å
*b* = 25.151 (3) Å
*c* = 8.8840 (11) Å
β = 98.010 (1)°
*V* = 1791.1 (4) Å^3^
*Z* = 4
Mo *K*α radiation
μ = 0.21 mm^−1^
*T* = 296 K
0.39 × 0.29 × 0.25 mm

#### Data collection

Bruker SMART CCD area-detector diffractometer
Absorption correction: multi-scan (*SADABS*; Sheldrick, 2003 ▶) *T*_min_ = 0.923, *T*_max_ = 0.949
9993 measured reflections
3336 independent reflections
2570 reflections with *I* > 2σ(*I*)
*R*_int_ = 0.024

#### Refinement

*R*[*F* ^2^ > 2σ(*F* ^2^)] = 0.039
*wR*(*F* ^2^) = 0.111
*S* = 1.04
3336 reflections
230 parameters
H-atom parameters constrained
Δρ_max_ = 0.35 e Å^−3^
Δρ_min_ = −0.37 e Å^−3^

### 

Data collection: *SMART* (Bruker, 2001 ▶); cell refinement: *SAINT* (Bruker, 2003 ▶); data reduction: *SAINT*; program(s) used to solve structure: *SHELXS97* (Sheldrick, 2008 ▶); program(s) used to refine structure: *SHELXL97* (Sheldrick, 2008 ▶); molecular graphics: *SHELXTL* (Sheldrick, 2008 ▶); software used to prepare material for publication: *SHELXTL*.

## Supplementary Material

Crystal structure: contains datablocks global, I. DOI: 10.1107/S1600536811011949/kp2315sup1.cif

Structure factors: contains datablocks I. DOI: 10.1107/S1600536811011949/kp2315Isup2.hkl

Additional supplementary materials:  crystallographic information; 3D view; checkCIF report

## Figures and Tables

**Table 1:** Hydrogen-bond geometry (Å, °) *Cg*1 is the centroid of the C1–C6 ring.

*D*—H⋯*A*	*D*—H	H⋯*A*	*D*⋯*A*	*D*—H⋯*A*
N1—H1⋯O1^i^	0.86	2.27	2.903 (2)	131
O2—H2*A*⋯O5^ii^	0.82	1.79	2.598 (2)	168
C16—H16*C*⋯*Cg*1	0.96	2.98	3.576 (3)	121

Symmetry codes: (i) 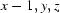; (ii) 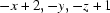.

**Fig. 1.**
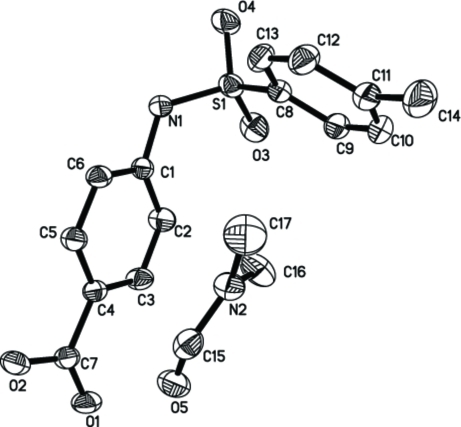
The ORTEP drawing of the title compound (I). Displacement ellipsoids are drawn at 30% probability level. All hydrogen atams have been omitted for clarity.

**Fig. 2.**
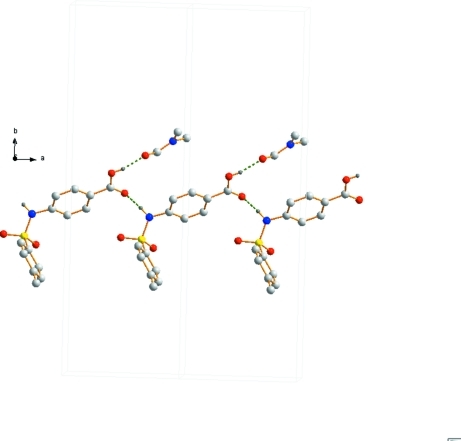
A view of the hydrogen bonds (dotted lines) in the crystal structure of the title compound (I).

**Fig. 3.**
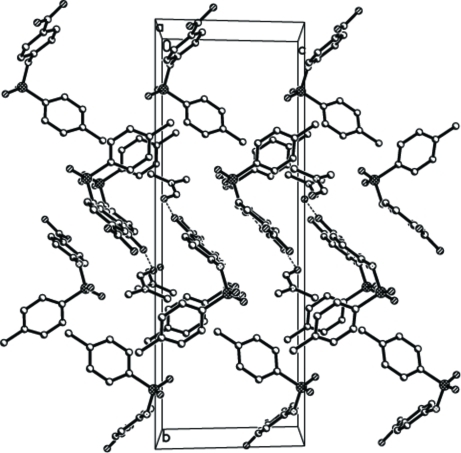
The crystal packing of the title compound (I), viewed along the c axis.

## supplementary crystallographic information

### Comment

N-Protected amino acids possess *R*-CONH-*R*' group analogous to the structure of O-terminal peptides and proteins (Menabue, *et al.*, 1988, Antolini, *et al.*, 1984). The substitution of an Ar—SO_2_-group on amine addes to 4-aminobenzeoic acid the coordination donors of three types-O, N donors from carboxyl, sulfoxyl and amine respectively, which may result in different coordination modes. In this paper, we synthesized the desired ligand in *N*,*N*-dimethylformamide solvent.

The title compound contains one *N*-*p*-tolysulfonyl-4-aminobenzoic acid molecule and a solvent N, *N*-dimethylformamide in the asymmetric unit (Fig. 1). The molecule has a C1—N1—S1—C8 of 64.552 (2) °, and the dihedral angle between the benzene rings is 83.367 (6) °. There are intermolecular hydrogen bonds between carboxylate group oxygen atoms, nitrogen atoms and solvent oxygen atoms of the types N—H···O and O—H···O generating a chain along *a* axis (Figs. 2 and 3, Table 1). There are intramolecular CH···π interactions between H(16 C) and H(17B) of solvent and the centre of two aromatic rings of the title molecule(C16—H16C···C1—C2—C3—C4—C5—C6 and C17—H17B···C8—C9—C10—C11—C12—C13; the distances of H to the centre of the aromatic rings are 2.982 (3) and 3.84 (3) Å, respectively).

### Experimental

A mixture of *N*-*p*-tolysulfonylchloride (1 mmol) and 4-aminobenzoic acid (1 mmol) in water(20 ml) was stirred at room temperature for 10 h. Then HCl (12 mol/*L*) was slowly added to the resulting solution. The mixture was stirred for 5 min and filtrated. The block colourless single crystals suitable for X-ray analysis were obtained by recrystallization from *N*,*N*-dimethylformamide. IR(KBr): 3423(*s*), 3198(*versus*), 3059(w), 2928(*s*), 2510(*m*), 1693(*versus*), 1635(*versus*), 1607(*s*), 1512(*m*), 1433(*m*), 1405(*s*), 1383(*s*), 1334(*s*), 1291(*s*), 1231(*m*), 1159(*versus*), 1092(*s*), 1021(w), 923(*s*), 862(*m*), 827(*m*), 765(*m*), 674(*s*), 5757(*s*), 547(*s*), 525(*m*), 503(w)cm^-1^.

### Refinement

H atoms were placed in calculated positions and treated as riding on their parent atoms (C—H = 0.93–0.96 Å, N—H = 0.86 Å,*O*—H = 0.82 Å) and *U*_iso_(H) = 1.2Ueq(C,*N*).

### Crystal data

**Table:**

C_14_H_13_NO_4_S·C_3_H_7_NO	*F*(000) = 768
*M**_r_* = 364.41	*D*_x_ = 1.351 Mg m^−^^3^
Monoclinic, *P*2_1_/*n*	Mo *K*α radiation, λ = 0.71073 Å
Hall symbol: -P 2yn	Cell parameters from 3230 reflections
*a* = 8.0953 (10) Å	θ = 2.5–25.0°
*b* = 25.151 (3) Å	µ = 0.21 mm^−^^1^
*c* = 8.8840 (11) Å	*T* = 296 K
β = 98.010 (1)°	Block, colourless
*V* = 1791.1 (4) Å^3^	0.39 × 0.29 × 0.25 mm
*Z* = 4	

### Data collection

**Table:**

Bruker SMART CCD area-detector diffractometer	3336 independent reflections
Radiation source: fine-focus sealed tube	2570 reflections with *I* > 2σ(*I*)
graphite	*R*_int_ = 0.024
phi and ω scans	θ_max_ = 25.5°, θ_min_ = 2.5°
Absorption correction: multi-scan (*SADABS*; Sheldrick, 2003)	*h* = −9→9
*T*_min_ = 0.923, *T*_max_ = 0.949	*k* = −23→30
9993 measured reflections	*l* = −10→10

### Refinement

**Table:**

Refinement on *F*^2^	Primary atom site location: structure-invariant direct methods
Least-squares matrix: full	Secondary atom site location: difference Fourier map
*R*[*F*^2^ > 2σ(*F*^2^)] = 0.039	Hydrogen site location: inferred from neighbouring sites
*wR*(*F*^2^) = 0.111	H-atom parameters constrained
*S* = 1.04	*w* = 1/[σ^2^(*F*_o_^2^) + (0.0517*P*)^2^ + 0.5453*P*] where *P* = (*F*_o_^2^ + 2*F*_c_^2^)/3
3336 reflections	(Δ/σ)_max_ < 0.001
230 parameters	Δρ_max_ = 0.35 e Å^−^^3^
0 restraints	Δρ_min_ = −0.37 e Å^−^^3^

### Special details

**Table:**

Geometry. All e.s.d.'s (except the e.s.d. in the dihedral angle between two l.s. planes)are estimated using the full covariance matrix. The cell e.s.d.'s are takeninto account individually in the estimation of e.s.d.'s in distances, anglesand torsion angles; correlations between e.s.d.'s in cell parameters are onlyused when they are defined by crystal symmetry. An approximate (isotropic)treatment of cell e.s.d.'s is used for estimating e.s.d.'s involving l.s. planes.
Refinement. Refinement of *F*^2^ against ALL reflections. The weighted *R*-factor *wR* and goodness of fit *S* are based on *F*^2^, conventional *R*-factors *R* are based on *F*, with *F* set to zero for negative *F*^2^. The threshold expression of *F*^2^ > σ(*F*^2^) is used only for calculating *R*-factors(gt) *etc*. and is not relevant to the choice of reflections for refinement. *R*-factors based on *F*^2^ are statistically about twice as large as those based on *F*, and *R*- factors based on ALL data will be even larger.

### Fractional atomic coordinates and isotropic or equivalent isotropic displacement parameters (Å^2^)

**Table:**

	*x*	*y*	*z*	*U*_iso_*/*U*_eq_	
C1	0.3556 (2)	0.05871 (7)	0.1256 (2)	0.0380 (4)	
C2	0.4907 (2)	0.07414 (9)	0.0562 (2)	0.0498 (5)	
H2	0.4753	0.0971	−0.0266	0.060*	
C3	0.6483 (2)	0.05551 (9)	0.1098 (2)	0.0488 (5)	
H3	0.7388	0.0665	0.0637	0.059*	
C4	0.6733 (2)	0.02077 (8)	0.2312 (2)	0.0400 (4)	
C5	0.5375 (2)	0.00633 (8)	0.3018 (2)	0.0449 (5)	
H5	0.5529	−0.0167	0.3846	0.054*	
C6	0.3800 (2)	0.02563 (8)	0.2509 (2)	0.0451 (5)	
H6	0.2905	0.0164	0.3008	0.054*	
C7	0.8422 (2)	−0.00147 (8)	0.2797 (2)	0.0452 (5)	
C8	0.1600 (2)	0.17188 (8)	0.1824 (2)	0.0459 (5)	
C9	0.2493 (3)	0.21904 (9)	0.1886 (3)	0.0560 (6)	
H9	0.2980	0.2302	0.1052	0.067*	
C10	0.2656 (3)	0.24944 (9)	0.3195 (3)	0.0632 (6)	
H10	0.3253	0.2811	0.3230	0.076*	
C11	0.1950 (3)	0.23365 (9)	0.4452 (3)	0.0602 (6)	
C12	0.1080 (3)	0.18598 (10)	0.4370 (3)	0.0625 (6)	
H12	0.0610	0.1745	0.5211	0.075*	
C13	0.0896 (3)	0.15518 (9)	0.3078 (3)	0.0555 (6)	
H13	0.0303	0.1234	0.3047	0.067*	
C14	0.2119 (4)	0.26663 (12)	0.5889 (4)	0.0878 (9)	
H14A	0.1492	0.2989	0.5700	0.132*	
H14B	0.3273	0.2750	0.6199	0.132*	
H14C	0.1699	0.2469	0.6679	0.132*	
C15	0.7728 (3)	0.09910 (9)	0.6138 (2)	0.0540 (6)	
H15	0.7882	0.0853	0.7119	0.065*	
C16	0.6101 (3)	0.15217 (11)	0.4239 (3)	0.0717 (7)	
H16A	0.7017	0.1442	0.3698	0.108*	
H16B	0.5986	0.1900	0.4319	0.108*	
H16C	0.5092	0.1375	0.3702	0.108*	
C17	0.5200 (4)	0.13996 (13)	0.6762 (4)	0.0936 (10)	
H17A	0.5620	0.1273	0.7761	0.140*	
H17B	0.4173	0.1221	0.6400	0.140*	
H17C	0.5005	0.1776	0.6802	0.140*	
N1	0.18945 (19)	0.07351 (7)	0.06580 (18)	0.0446 (4)	
H1	0.1149	0.0489	0.0558	0.054*	
N2	0.6410 (2)	0.12933 (7)	0.5741 (2)	0.0518 (4)	
O1	0.96596 (17)	0.01262 (6)	0.22823 (18)	0.0589 (4)	
O2	0.84373 (17)	−0.03862 (7)	0.38401 (19)	0.0628 (4)	
H2A	0.9385	−0.0505	0.4047	0.094*	
O3	0.2361 (2)	0.15441 (7)	−0.08607 (17)	0.0661 (5)	
O4	−0.04468 (18)	0.12891 (6)	−0.03477 (18)	0.0621 (4)	
O5	0.87652 (19)	0.08779 (7)	0.53101 (19)	0.0625 (4)	
S1	0.13037 (6)	0.13338 (2)	0.01515 (6)	0.04889 (17)	

### Atomic displacement parameters (Å^2^)

**Table:**

	*U*^11^	*U*^22^	*U*^33^	*U*^12^	*U*^13^	*U*^23^
C1	0.0357 (10)	0.0379 (10)	0.0400 (10)	0.0011 (8)	0.0036 (8)	−0.0049 (8)
C2	0.0447 (12)	0.0610 (14)	0.0444 (11)	0.0025 (10)	0.0084 (9)	0.0134 (10)
C3	0.0369 (11)	0.0612 (14)	0.0501 (12)	−0.0020 (9)	0.0124 (9)	0.0063 (10)
C4	0.0336 (10)	0.0438 (11)	0.0424 (10)	−0.0017 (8)	0.0044 (8)	−0.0040 (9)
C5	0.0387 (11)	0.0504 (12)	0.0459 (11)	0.0025 (9)	0.0073 (8)	0.0092 (9)
C6	0.0348 (11)	0.0506 (12)	0.0517 (12)	0.0004 (9)	0.0122 (9)	0.0067 (9)
C7	0.0353 (10)	0.0476 (12)	0.0522 (12)	−0.0024 (9)	0.0046 (8)	−0.0041 (9)
C8	0.0364 (11)	0.0419 (12)	0.0582 (12)	0.0028 (8)	0.0025 (9)	0.0042 (9)
C9	0.0480 (13)	0.0471 (13)	0.0736 (15)	−0.0006 (10)	0.0112 (11)	0.0114 (11)
C10	0.0536 (14)	0.0423 (13)	0.0934 (18)	−0.0078 (10)	0.0091 (13)	−0.0037 (12)
C11	0.0488 (13)	0.0527 (14)	0.0794 (16)	−0.0030 (11)	0.0103 (11)	−0.0146 (12)
C12	0.0592 (14)	0.0635 (15)	0.0682 (15)	−0.0128 (12)	0.0207 (11)	−0.0104 (12)
C13	0.0540 (13)	0.0482 (13)	0.0659 (14)	−0.0130 (10)	0.0142 (11)	−0.0056 (11)
C14	0.083 (2)	0.0776 (19)	0.106 (2)	−0.0165 (16)	0.0240 (17)	−0.0388 (17)
C15	0.0571 (14)	0.0541 (14)	0.0479 (12)	−0.0114 (11)	−0.0028 (10)	0.0104 (10)
C16	0.0728 (17)	0.0799 (18)	0.0596 (14)	0.0280 (14)	−0.0006 (12)	0.0042 (13)
C17	0.091 (2)	0.102 (2)	0.100 (2)	0.0006 (17)	0.0533 (18)	−0.0015 (18)
N1	0.0352 (9)	0.0432 (10)	0.0532 (10)	−0.0005 (7)	−0.0017 (7)	−0.0013 (7)
N2	0.0465 (10)	0.0578 (11)	0.0517 (10)	0.0023 (8)	0.0088 (8)	0.0015 (8)
O1	0.0336 (8)	0.0704 (11)	0.0740 (10)	−0.0024 (7)	0.0121 (7)	0.0075 (8)
O2	0.0371 (8)	0.0679 (11)	0.0827 (11)	0.0070 (7)	0.0059 (8)	0.0237 (9)
O3	0.0747 (11)	0.0672 (11)	0.0587 (9)	0.0091 (8)	0.0173 (8)	0.0190 (8)
O4	0.0481 (9)	0.0665 (11)	0.0659 (10)	0.0111 (7)	−0.0123 (7)	0.0033 (8)
O5	0.0456 (9)	0.0688 (11)	0.0723 (10)	0.0096 (7)	0.0055 (8)	0.0127 (8)
S1	0.0447 (3)	0.0511 (3)	0.0492 (3)	0.0066 (2)	0.0007 (2)	0.0071 (2)

### Geometric parameters (Å, °)

**Table:**

C1—C6	1.381 (3)	C12—C13	1.375 (3)
C1—C2	1.384 (3)	C12—H12	0.9300
C1—N1	1.425 (2)	C13—H13	0.9300
C2—C3	1.380 (3)	C14—H14A	0.9600
C2—H2	0.9300	C14—H14B	0.9600
C3—C4	1.380 (3)	C14—H14C	0.9600
C3—H3	0.9300	C15—O5	1.224 (3)
C4—C5	1.388 (2)	C15—N2	1.317 (3)
C4—C7	1.485 (3)	C15—H15	0.9300
C5—C6	1.380 (3)	C16—N2	1.442 (3)
C5—H5	0.9300	C16—H16A	0.9600
C6—H6	0.9300	C16—H16B	0.9600
C7—O1	1.211 (2)	C16—H16C	0.9600
C7—O2	1.315 (2)	C17—N2	1.450 (3)
C8—C13	1.386 (3)	C17—H17A	0.9600
C8—C9	1.386 (3)	C17—H17B	0.9600
C8—S1	1.762 (2)	C17—H17C	0.9600
C9—C10	1.382 (3)	N1—S1	1.6243 (17)
C9—H9	0.9300	N1—H1	0.8600
C10—C11	1.382 (3)	O2—H2A	0.8200
C10—H10	0.9300	O3—S1	1.4266 (16)
C11—C12	1.387 (3)	O4—S1	1.4291 (15)
C11—C14	1.512 (3)		
			
C6—C1—C2	119.62 (17)	C12—C13—H13	120.3
C6—C1—N1	118.66 (16)	C8—C13—H13	120.3
C2—C1—N1	121.62 (17)	C11—C14—H14A	109.5
C3—C2—C1	120.08 (18)	C11—C14—H14B	109.5
C3—C2—H2	120.0	H14A—C14—H14B	109.5
C1—C2—H2	120.0	C11—C14—H14C	109.5
C2—C3—C4	120.81 (18)	H14A—C14—H14C	109.5
C2—C3—H3	119.6	H14B—C14—H14C	109.5
C4—C3—H3	119.6	O5—C15—N2	124.8 (2)
C3—C4—C5	118.68 (17)	O5—C15—H15	117.6
C3—C4—C7	119.61 (17)	N2—C15—H15	117.6
C5—C4—C7	121.68 (18)	N2—C16—H16A	109.5
C6—C5—C4	120.87 (18)	N2—C16—H16B	109.5
C6—C5—H5	119.6	H16A—C16—H16B	109.5
C4—C5—H5	119.6	N2—C16—H16C	109.5
C5—C6—C1	119.87 (17)	H16A—C16—H16C	109.5
C5—C6—H6	120.1	H16B—C16—H16C	109.5
C1—C6—H6	120.1	N2—C17—H17A	109.5
O1—C7—O2	123.18 (18)	N2—C17—H17B	109.5
O1—C7—C4	123.84 (19)	H17A—C17—H17B	109.5
O2—C7—C4	112.97 (16)	N2—C17—H17C	109.5
C13—C8—C9	119.9 (2)	H17A—C17—H17C	109.5
C13—C8—S1	119.26 (16)	H17B—C17—H17C	109.5
C9—C8—S1	120.86 (17)	C1—N1—S1	124.87 (13)
C10—C9—C8	119.7 (2)	C1—N1—H1	117.6
C10—C9—H9	120.2	S1—N1—H1	117.6
C8—C9—H9	120.2	C15—N2—C16	120.48 (18)
C11—C10—C9	121.2 (2)	C15—N2—C17	122.1 (2)
C11—C10—H10	119.4	C16—N2—C17	117.4 (2)
C9—C10—H10	119.4	C7—O2—H2A	109.5
C10—C11—C12	118.0 (2)	O3—S1—O4	119.31 (10)
C10—C11—C14	121.7 (2)	O3—S1—N1	109.82 (9)
C12—C11—C14	120.3 (2)	O4—S1—N1	104.71 (9)
C13—C12—C11	121.7 (2)	O3—S1—C8	107.75 (10)
C13—C12—H12	119.1	O4—S1—C8	108.51 (9)
C11—C12—H12	119.1	N1—S1—C8	106.00 (9)
C12—C13—C8	119.4 (2)		
			
C6—C1—C2—C3	1.5 (3)	C10—C11—C12—C13	−0.8 (4)
N1—C1—C2—C3	−174.66 (19)	C14—C11—C12—C13	179.6 (2)
C1—C2—C3—C4	1.0 (3)	C11—C12—C13—C8	0.3 (4)
C2—C3—C4—C5	−2.2 (3)	C9—C8—C13—C12	0.6 (3)
C2—C3—C4—C7	175.86 (19)	S1—C8—C13—C12	−177.99 (18)
C3—C4—C5—C6	0.9 (3)	C6—C1—N1—S1	135.20 (17)
C7—C4—C5—C6	−177.16 (19)	C2—C1—N1—S1	−48.6 (2)
C4—C5—C6—C1	1.7 (3)	O5—C15—N2—C16	−0.3 (4)
C2—C1—C6—C5	−2.9 (3)	O5—C15—N2—C17	178.1 (2)
N1—C1—C6—C5	173.46 (18)	C1—N1—S1—O3	51.58 (18)
C3—C4—C7—O1	7.1 (3)	C1—N1—S1—O4	−179.18 (15)
C5—C4—C7—O1	−174.9 (2)	C1—N1—S1—C8	−64.55 (17)
C3—C4—C7—O2	−172.33 (19)	C13—C8—S1—O3	−170.90 (16)
C5—C4—C7—O2	5.7 (3)	C9—C8—S1—O3	10.6 (2)
C13—C8—C9—C10	−0.8 (3)	C13—C8—S1—O4	58.62 (19)
S1—C8—C9—C10	177.71 (17)	C9—C8—S1—O4	−119.92 (17)
C8—C9—C10—C11	0.2 (3)	C13—C8—S1—N1	−53.38 (19)
C9—C10—C11—C12	0.6 (4)	C9—C8—S1—N1	128.09 (17)
C9—C10—C11—C14	−179.9 (2)		

### Hydrogen-bond geometry (Å, °)

**Table:**

Cg1 is the centroid of the C1–C6 ring.

**Table:**

*D*—H···*A*	*D*—H	H···*A*	*D*···*A*	*D*—H···*A*
N1—H1···O1^i^	0.86	2.27	2.903 (2)	131
O2—H2A···O5^ii^	0.82	1.79	2.598 (2)	168
C16—H16C···Cg1	0.96	2.98	3.576 (3)	121

Symmetry codes: (i) *x*−1, *y*, *z*; (ii) −*x*+2, −*y*, −*z*+1.
